# Anti-cancer Drug Response Prediction Using Neighbor-Based Collaborative Filtering with Global Effect Removal

**DOI:** 10.1016/j.omtn.2018.09.011

**Published:** 2018-09-22

**Authors:** Hui Liu, Yan Zhao, Lin Zhang, Xing Chen

**Affiliations:** 1 School of Information and Control Engineering, China University of Mining and Technology, Xuzhou, China

**Keywords:** drug response, cancer, prediction, collaborative filtering, global effect

## Abstract

Patients of the same cancer may differ in their responses to a specific medical therapy. Identification of predictive molecular features for drug sensitivity holds the key in the era of precision medicine. Human cell lines have harbored most of the same genetic changes found in patients’ tumors and thus are widely used in the research of drug response. In this work, we formulated drug-response prediction as a recommender system problem and then adopted a neighbor-based collaborative filtering with global effect removal (NCFGER) method to estimate anti-cancer drug responses of cell lines by integrating cell-line similarity networks and drug similarity networks based on the fact that similar cell lines and similar drugs exhibit similar responses. Specifically, we removed the global effect in the available responses and shrunk the similarity score for each cell line pair as well as each drug pair. We then used the K most similar neighbors (hybrid of cell-line-oriented and drug-oriented) in the available responses to predict the unknown ones. Through 10-fold cross-validation, this approach was shown to reach accurate and reproducible outcomes of drug sensitivity. We also discussed the biological outcomes based on the newly predicted response values.

## Graphical Abstract

**Figure:**
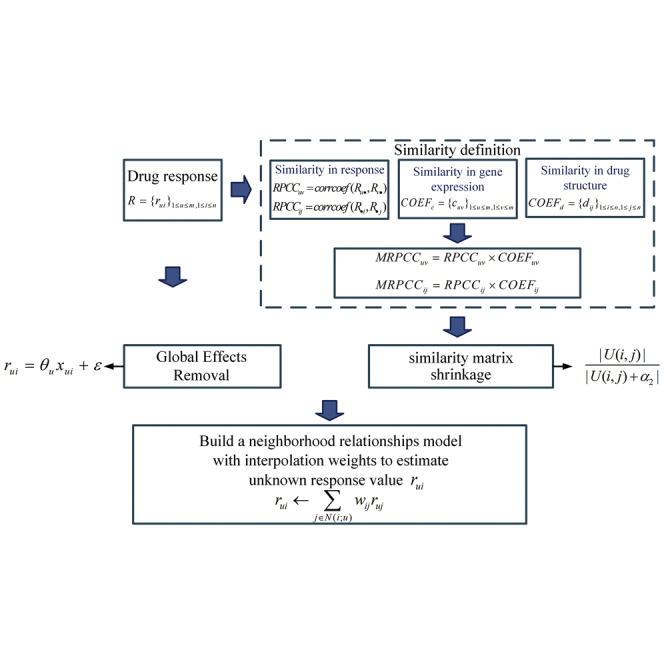

## Introduction

Cancer subtypes differ in chemotherapeutic response and thus may require different medical treatment. The relationships between molecular features and clinical drug responses lay the foundation for optimizing drug therapies based on a patient’s genomic context.^1^ Therefore, it has been a major challenge to accurately predict the anti-cancer drug response based on the patient’s molecular and clinical profiles in the era of precision medicine. On the one hand, it is crucial for clinicians to make decisions in the choice of most effective and least toxic therapeutic regimen. On the other hand, the identification of a drug-sensitive biomarker is essential for cancer medicine. The emerging of high-throughput drug-screening technologies has enabled many studies to conduct large-scale experiments on cultured human cell line panels, which greatly improved systematical elucidation of the response mechanism of anti-cancer drugs. Several attempts to construct predictive models for drug response have made use of some datasets. For instance, NCI-60 was a panel of human cell lines originally derived from human cancers spanning nine different tissues of origin.^2^ The other two recent consortiums, GDSC (Genomics of Drug Sensitivity in Cancer)^3^ and CCLE (Cancer Cell Line Encyclopedia)^4^ have analyzed around 1,500 cancer cell lines and their genomic profiles against 280 drugs, providing the concentration required for 50% of cellular growth inhibition (IC^50^) or activity area as drug-response measurement. All studies provide genome-wide profiling of multiple cancer cell lines and drug-sensitivity data based on established anti-cancer drugs against the cell lines. However, the sensitivity levels for most cell line-drug pairs are still unknown, and it needs to be achieved by a time- and cost-effective way for potential personalized medicine.^5^

Currently, most commonly used methods for drug response prediction are multivariate linear regression (least absolute shrinkage and selection operator [LASSO] and elastic net regularizations) and nonlinear regression (e.g., neural networks and some kernel-based methods).^3^, ^4^, ^6^, ^7^, ^8^, ^9^ Daemen et al.^10^ identified response-associated molecular features, such as measurements of copy number aberrations, mutations, gene and isoform expression, promoter methylation, as well as protein expression in breast cancer by least-squares-support vector machines and random forest algorithms. Staunton et al.^11^ first developed a weighted voting classification model on NCI-60 basal gene expression data to predict anti-cancer drug sensitivity. Gene signatures of 232 drugs from 6,817 genes were created to predict a binary (sensitive or resistant) response. Riddick et al.^12^ built an ensemble regression model using random forest to predict *in vitro* drug response from a signature of basal-gene expression. The random forest regression model for each drug was built between gene-expression signatures for the cell lines and the corresponding IC^50^ values for the exact drug for unknown response prediction. Cortes-Ciriano et al.^13^ proposed a simultaneous machine-learning modeling of chemical and cell-line information for response prediction. Ammad-ud-din et al.^14^ adopted a kernelized Bayesian matrix factorization (KBMF) method to predict the drug responses by integrating genomic and chemical properties in addition to drug target information. Zhang et al.^15^ proposed a dual-layer cell-line drug network (DLN) model, which integrated both cell-line similarity network data and drug similarity network data, to predict the missing drug response of a given cell line. Kim et al.^16^ developed a network-based classifier method for predicting sensitivity of cell lines to anti-cancer drugs from transcriptome data. Wang et al.^17^ proposed a similarity-regularized matrix factorization (SRMF) method for drug-response prediction, which incorporates similarities of drugs and of cell lines simultaneously. Stanfield et al.^18^ proposed a heterogeneous network-based method to predict the interaction between cell line-drug pairs. They classified the interaction between each cell line-drug pairs into sensitive and resistant and thus turned the prediction problem into classification. Suphavilai et al. have proposed a matrix factorization based recommender system (CaDRReS) method, which considers essential genes for drug-response prediction.^19^

Regarding the fact that similar cell lines and similar drugs exhibit similar drug responses,^15^ the prediction of unknown drug response can be considered as a typical recommender system (RS).^20^ Typically, in a RS, there is a set of users and a set of items. Each user *u* rates a set of items by some values. The RS attempts to profile user preferences and tries to model the interaction between users and items, which is exactly analog for drug-response prediction. The cell lines correspond to users while drugs correspond to items. Thus, we proposed an RS technique, neighborhood-based collaborative filtering with global effects removal (NCFGER), for drug-response prediction, which incorporates similarities of drugs and of cell lines in additional to the known drug response simultaneously. To demonstrate its effectiveness, we compared NCFGER with SRMF, which has been proved to show higher performance than typical similarity-based methods KBMF and DLN. The evaluation metrics are also averaged Pearson correlation coefficient (PCC) and averaged root-mean-square error (RMSE) over all drugs. The results on GDSC and CCLE drug-response datasets by 10-fold cross validation showed that NCFGER performed dramatically better than SRMF in terms of drug-averaged PCC and RMSE. NCFGER has also been applied to impute unknown response values in the GDSC dataset for detailed biological meaningful presentation.

## Results

### Measurements of Prediction Performance

The prediction performance of our method was evaluated using PCC and RMSE between predicted and observed drug responses for each drug. A higher PCC and lower RMSE indicate a better prediction performance of a method. For comparison, PCC and RMSE of the sensitive and resistant cell lines of each individual drug, which were denoted as PCC_S/R (PCC between predicted and observed responses of sensitive and resistant cell lines) and RMSE_S/R (RMSE between predicted and observed responses of sensitive and resistant cell lines) were also evaluated, respectively. For each drug, the logIC^50^ values were split into quantiles, with cell lines in the first and fourth representing drug-sensitive (-resistant) and -resistant (-sensitive) cell lines, respectively, which followed the same definition in Wang et al.^17^ Therefore, we evaluated four measures, drug-averaged PCC, drug-averaged RMSE, drug-averaged PCC_S/R, as well as drug-averaged RMSE_S/R over all drugs, respectively.

### Similarity in Response Helps Improve the Prediction Performance

We first conducted 10-fold cross-validation to evaluate the prediction performance in the GDSC (https://www.cancerrxgene.org/) and CCLE (https://www.broadinstitute.org/ccle) datasets to evaluate different similarity definition. For each dataset, the drug-response entries were divided into 10 folds randomly with almost the same size. Each time, 1 fold was used as the test set, while the remaining 9 folds were used as the training sets. The prediction was repeated 10 times such that each fold acted as a test set once. The whole cross-validation was run 100 times for each dataset, and the prediction performance was compared with the best state-of-the-art method, SRMF.

As is shown in Tables S1 and S2, the performance of hybrid NCFGER with *RPCC* and *MRPCC* were better than that of *COEF* similarity, which indicates that the similarity exhibited in drug-response values can better improve the drug-response prediction performance. It was consistent with the result concluded in L.Z (unpublished data). Thus, we used *MRPCC* similarity in the following study.

### 10-Fold Cross-Validation Test on GDSC and CCLE Drug-Response Datasets

We also conducted 10-fold cross-validation on GDSC and CCLE drug-response datasets to investigate the performance of cell-line-based NCFGER, drug-based NCFGER, and hybrid NCFGER.

Tables 1 and S3 showed the comparison results obtained by four methods, hybrid NCFGER, cell-line-based NCFGER, drug-based NCFGER, and SRMF in the GDSC and CCLE datasets. As shown in Table 1, all NCFGER methods outperformed SRMF in all metrics in the GDSC dataset, while hybrid NCFGER attains the best performance. The drug-averaged PCC_S/R obtained by our method is 0.81, which is 14.42% higher than that of SRMF. The drug-averaged RMSE_S/R obtained by our method is 1.42, which is 17.92% lower than that obtained by SRMF.

**Table 1:** Comparisons of Both Methods Obtained under 10-Fold Cross-Validation on GDSC Dataset

Methods	Drug-Averaged PCC_S/R	Drug-Averaged RMSE_S/R	Drug-Averaged PCC	Drug-Averaged RMSE
**NCFGER**				

Hybrid	0.81 (±0.11)	1.42 (±0.29)	0.73 (±0.13)	1.18 (±0.24)
Cell-line based	0.76 (±0.14)	1.49 (±0.24)	0.67 (±0.15)	1.29 (±0.21)
Drug-based	0.75 (±0.12)	1.60 (±0.43)	0.66 (±0.14)	1.32 (±0.34)

**SRMF**				

	0.71 (±0.15)	1.73 (±0.46)	0.62 (±0.16)	1.43 (±0.36)

As shown in Table S3, all NCFGER methods also outperform SRMF in all metrics in the CCLE dataset, and hybrid NCFGER still attains the best performance. The drug-averaged PCC_S/R obtained by our method is 0.89, which is 14.40% higher than SRMF. The drug-averaged RMSE_S/R obtained by our method is 0.47, which is 36.08% lower than that obtained by SRMF.

Considering the results drawn in above, we focused on hybrid NCFGER for further analysis if there was no additional explanation. The cell-line-based NCFGER method depended on the most similar *K* drugs that had responses in the same cell line, which means the drug structure contributed to the prediction performance improvement from the respect of RS. Since neither cell-line-based nor drug-based NCFGER could outperform the hybrid method, both cell-line gene expression profile and drug structure contributed to the prediction performance improvement. If we only use one part of them, we will miss some information that helps to predict the drug responses.

**Figure 1:**
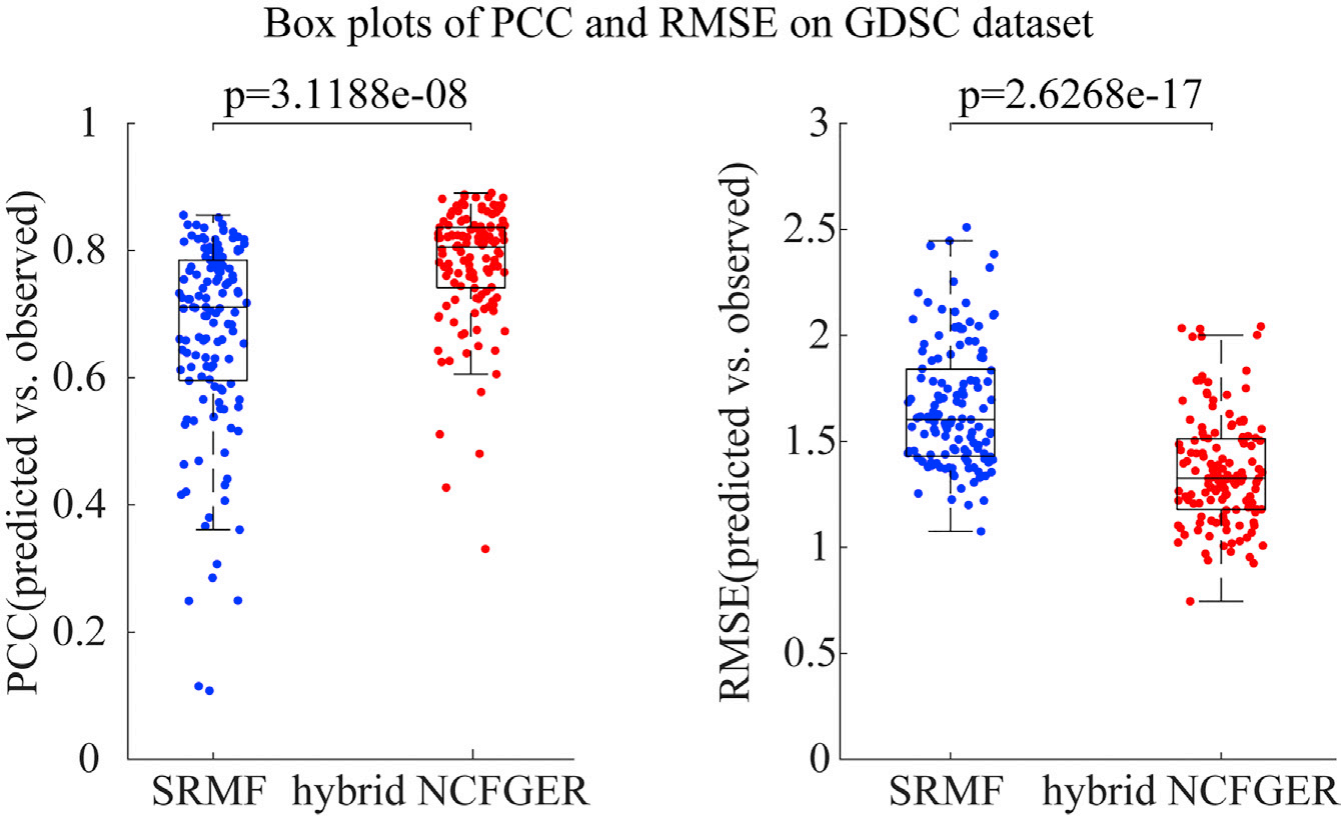
Boxplots of PCC and RMSE Based on SRMF and NCFGER

Figures 1 and S1 also show the boxplots of both methods with respect to PCC and RMSE for each drug in the CCLE and GDSC datasets, respectively. Either PCC or RMSE averaged for each drug over the 100 times of cross-validation from hybrid NCFGER is better than that of SRMF.

**Figure 2:**
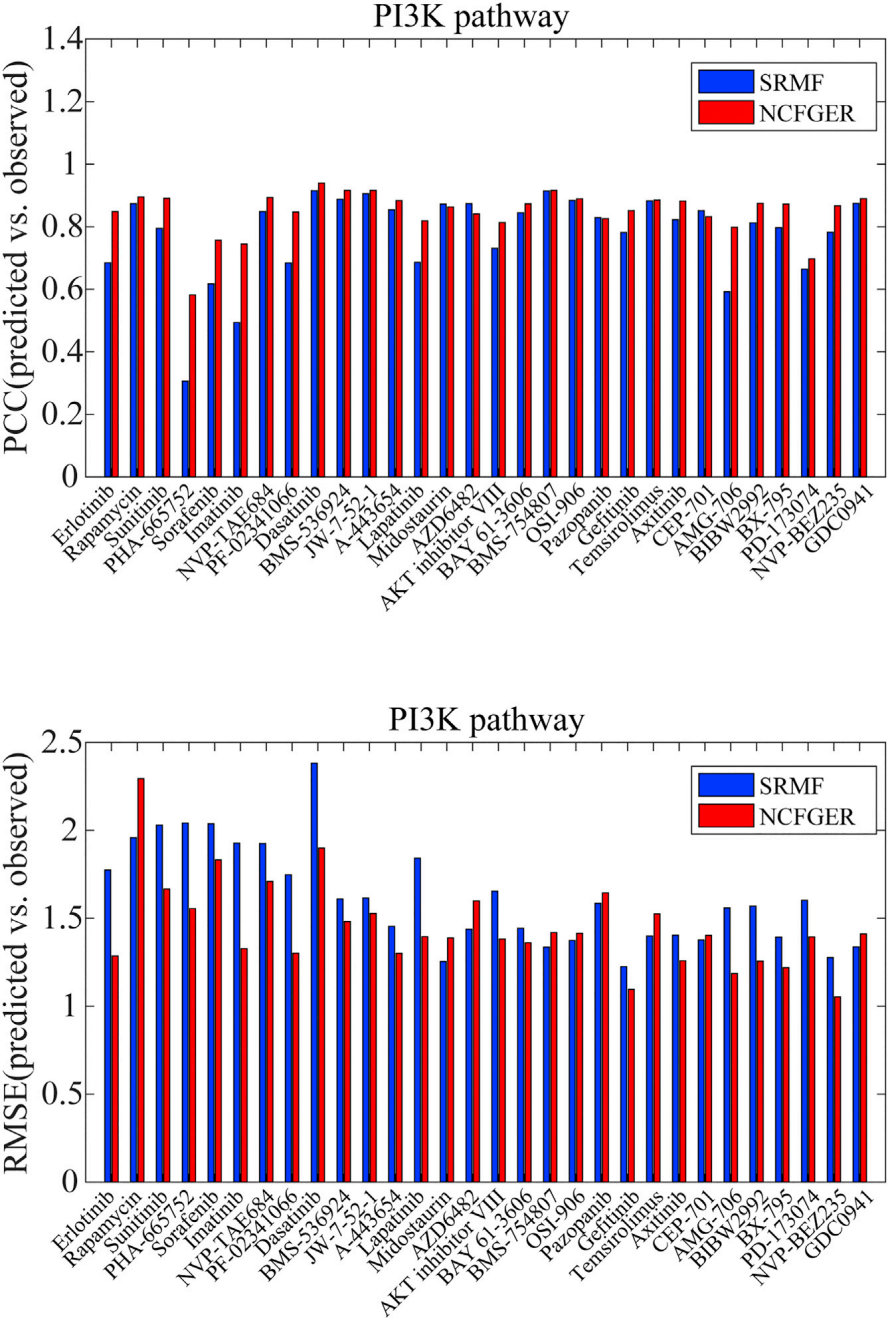
Prediction Performance Comparisons of SRMF and NCFGER for Drug-Targeting Genes in PI3K Pathway with Respect to PCC as Well as RMSE in CCLE Dataset

Furthermore, we also investigated the prediction performance of drug target genes in specific pathways. As is known, phosphatidylinositol 3-kinase (PI3K) and extracellular signal-related kinase (ERK) signaling pathways have been identified as promising therapeutic targets for cancer therapy, which makes it meaningful to consider the prediction performance of drug responses for their targeting genes in these pathways^21^ (Figures 2 and S2). From the perspective of PCC and RMSE, NCFGER performs better than SRMF in both PI3K and ERK pathways.

### Drug-Cancer Association Validation Based on Predicted Responses in GDSC Dataset

**Figure 3:**
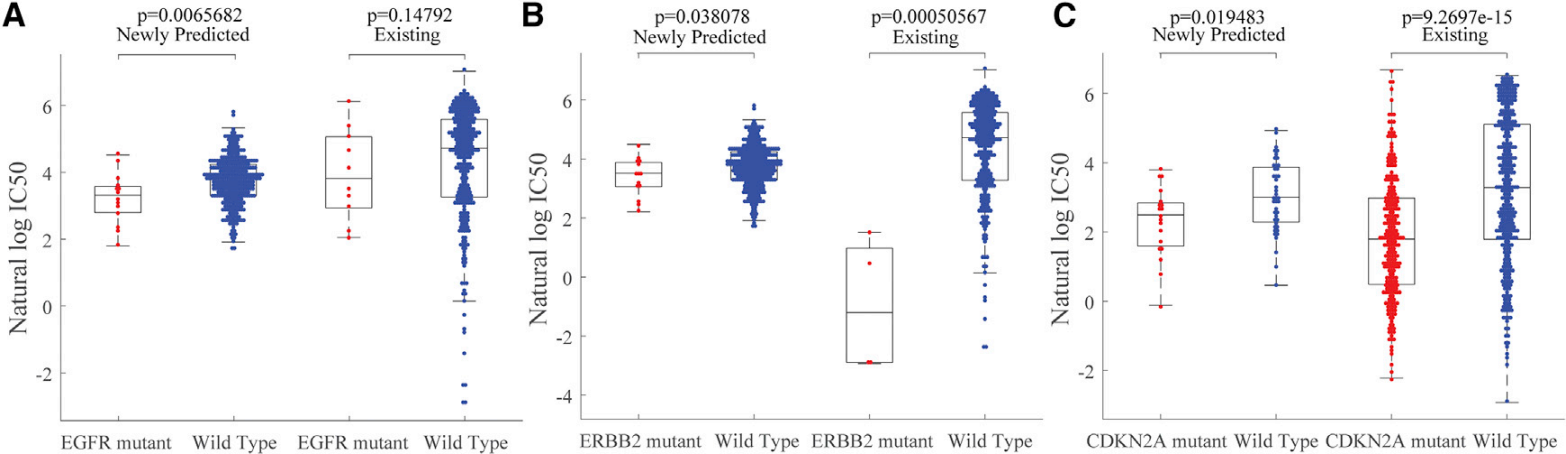
The Association of Drug Sensitivity and Cancer Gene Mutations Were Consistent for Predicted Response Data EGFR (A) and ERBB2 (B) mutated cell lines were more sensitive to the drug lapatinib, while CDKN2A (C) mutated cell lines were more sensitive to drug PD-0332991.

Using our proposed method mentioned in the above sections, we trained NCFGER method on all available response IC^50^ and used it to predict the missing responses in GDSC dataset. Following Wang et al.,^17^ we focused on an epidermal growth factor receptor (EGFR)-family inhibitor, lapatinib, where more than half of response values (342/652) were missing and a cyclin-dependent kinases (CDKs) 4 and 6 inhibitor PD-0332991, where nearly 10% of response values (62/652) were missing. Based on the definition that a mutated gene fulfills any of these criteria following Garnett et al.^7^: a coding sequence variant in the cancer gene, a total copy number = 0 (homozygous deletion) or ≥ 8 (amplification), we grouped the unassayed cell lines based on their EGFR mutation profiles and found that the EGFR mutated cell lines were significantly more sensitive to lapatinib. EGFR and ERBB2 amplification was shown to be associated with sensitivity to lapatinib, which has been licensed for the treatment of HER2-positive breast cancer clinically^22^, ^23^ (Figure 3A). This prediction happened to coincide with that in assayed cell lines. Similar fact was observed with predicted response of ERBB2-mutated cell lines to lapatinib (Figure 3B), which is exactly consistent with previous literatures.^24^ As to PD-0332991, it is an inhibitor of upstream cyclin-dependent kinases (CDKs) 4 and 6, while CDKN2A-mutated cells have been known to have enhanced requirement for signaling through the CDK4/6-pRb signaling pathway. The predicted results show that CDKN2A-mutated cell lines were more sensitive to PD-0332991 (Figure 3C). This prediction was not only consistent with that in assayed cell lines, but also in agreement with previously a published study.

**Figure 4:**
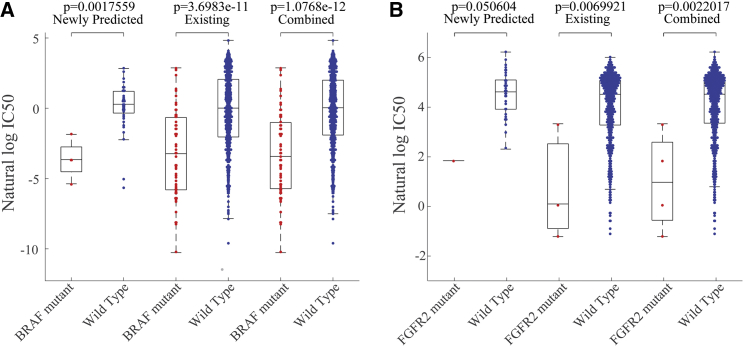
The New Drug-Cancer Association Was Also Observed Based on the Combination of Observed Responses and Newly Predicted Responses The new drug-cancer association was also observed based on the combination of observed responses and newly predicted responses. (A) BRAF is found to be more significantly associated with PD-0325901 with a combination of newly predicted and known responses. (B) FGFR2 is also found to be more significantly associated with PD-173074.

The newly predicted drug responses combined with existing drug responses were able to detect novel drug-cancer gene associations as well, which is consistent with previous literatures (Figure 4). For example, the oncogene BRAF has been found significantly associated with enhanced and selective sensitivity to mitogen-activated protein kinase (MEK) inhibitor PD-0325901 (p = 3.70e-11 for available responses; p = 1.08e-12 for combination of predicted and available responses),^25^ which was approached with the combination of newly predicted drug responses and known responses versus available responses themselves. Therefore, it is important to complete the unknown observations of drug response matrix such that we can unveil the new drug-sensitivity mechanism better. Also, based on the combined newly predicted drug responses and available observations versus available observations themselves, fibroblast growth factor receptor 2 (FGFR2)-mutated cell lines were exquisitely sensitive to PD-173074, which has been known to prevent signaling, at nanomolar levels, through FGFR2–5 (p = 0.7e-2 for available observations; p = 0.22e-2 for combination of predicted and available observations).^26^, ^27^, ^28^, ^29^

**Figure 5:**
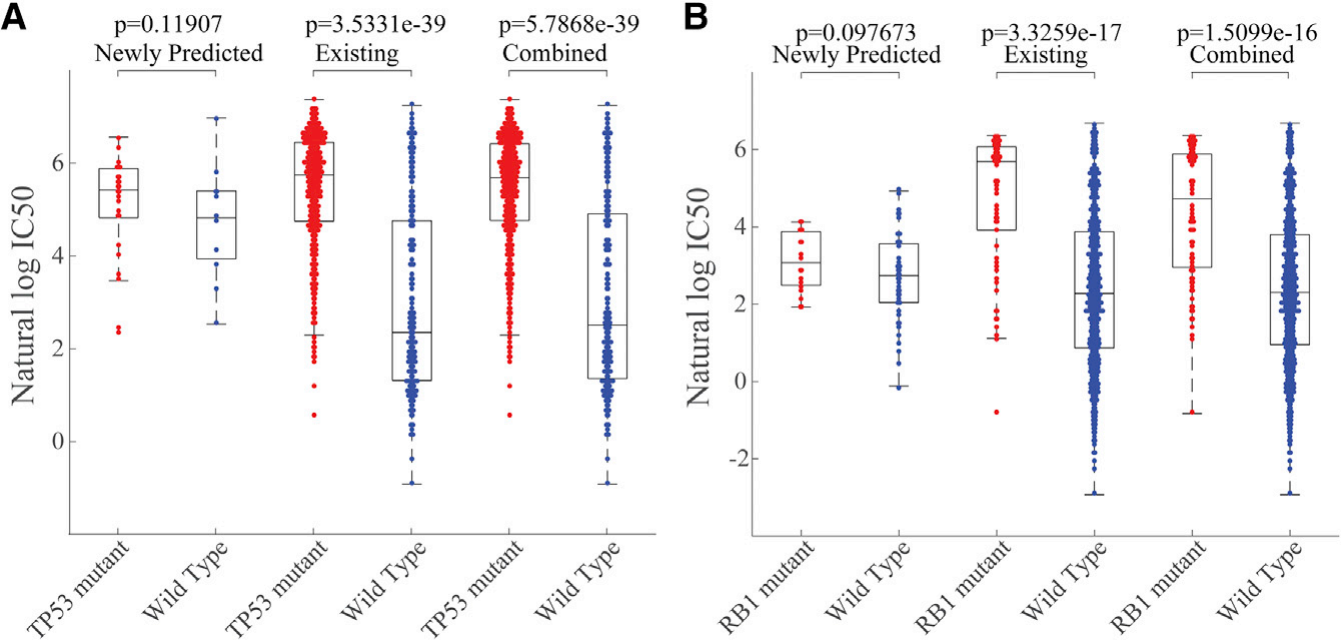
The Association between Inactivating Mutations in Tumor-Suppressor Genes and Drugs Were Also Obtained Based on the Combination of Observed Responses and Newly Predicted Responses The association between inactivating mutations in tumor suppressor genes and drugs were also obtained based on the combination of observed responses and newly predicted responses. (A) TP53 is found to be significantly resistant to Nutlin-3a based on the combination of known and unknown response. (B) RB1 is also found to be significantly resistant to PD-0332991.

The association between the presence of inactivating mutations in tumor-suppressor genes and several drugs, which in some aspect provide insight into the interaction between tumor suppressors and the cellular mechanism in mediating drug sensitivity, were also remained in the predicted drug response (Figure 5). For instance, mutation of TP53, an important regulator of apoptosis and cell cycle arrest in response to cellular stress, confers resistance to Nutlin-3a (p = 3.53e-39 for known response; p = 5.79e-39 for combination of known and unknown response), which is an inhibitor of the mouse double minute 2 homolog (MDM2) E3-ligase that negatively regulates p53 protein levels. Just like TP53, mutation inactivation of RB1, a key repressor of cell cycle progression in normal cells, confers resistance to PD-0332991 (p = 3.33e-17 for known response; p = 5.51e-16 for combination of known and unknown response).

**Figure 6:**
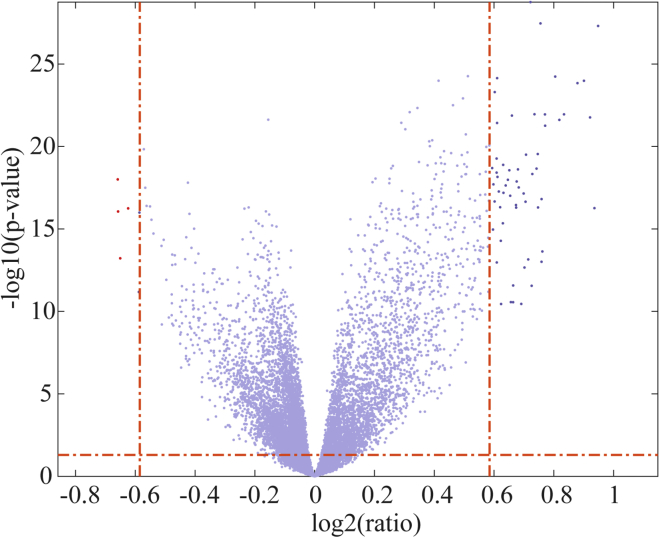
Volcano Plot of Gene Expression Profiles in Sunitinib-Sensitive and -Resistant Cell Lines

We also investigated the genes that are significantly differentially expressed in sensitive and resistant cell lines, as shown in Figure 6. The threshold p value was set to 0.05, and the threshold fold change was set to 1.5. Significantly differentially expressed genes further went through gene ontology enrichment analysis by DAVID^30^ (https://david.ncifcrf.gov/) with default parameter settings. Finally, differentially expressed genes were found to be related to the PI3K-Akt signaling pathway (p = 7.9e-4), ECM-receptor interaction (p = 3.3e-10), and small-cell lung cancer (p = 1.5e-4).

## Discussion

In this study, we developed a collaborative filtering-based method, NCFGER, to estimate the response of cancer cell lines to drug treatments for IC_50_ values as well as activity area in GDSC and CCLE datasets, respectively. This method is the hybrid of cell line-based and drug-based collaborative filtering techniques, thereby incorporating similarity in responses from the perspective of cell lines and drug structures, similarity in cell-line gene expression profile, as well as similarity in drug chemical fingerprint. It also applies a global effect removal to preprocess the available drug response and a shrinkage operation on both cell-line similarity network and drug similarity network to avoid the bias caused by unknown responses. 10-fold cross-validation showed that the drug-response similarity can better improve the drug-response prediction performance in comparison with the cell-line gene expression and drug chemical structure similarity. 10-fold cross-validation also showed that the hybrid NCFGER algorithm consistently outperformed SRMF, suggesting that the hybrid NCFGER are more predictive of anti-cancer drug response. We also used the hybrid NCFGER to predict the unknown drug-response values in the GDSC dataset. In combination with the available observations, the results of gene mutation association with drug response, inactivating mutation association in tumor gene suppressors with drugs were all consistent with previous findings.

Compared to the existing drug-response prediction method, our NCFGER method is based on the relatively dependable neighbors (both cell lines and drugs) and dependable similarity score shrinkage technique during response prediction, so the sparsity has less influence on the prediction performance. Furthermore, the prediction performance was not seriously affected by either similarity of cell-line gene expression or of drug; thus, the input for NCFGER could be quite simple.

Despite these encouraging aspects, NCFGER suffers from the following limitations, which we hope to address in the future. First, from the respect of the cell line, construction of NCFGER relies on gene-expression profile data only, and we hope to integrate somatic mutation information and epigenetic status in the future. Some pathway-related information or other dynamic information may also help improve the predictive performance of drug response; thus, it might be better to integrate omics data later. The neighbor-based collaborative filtering framework highly depends on the selection of neighbors. We may start the incorporation of other information, such as pathway-related information or other dynamic information, with the integration of this information for neighbor selection at the first step. We can also treat the drug-response prediction as a classification problem, which would be easier to incorporate the other available information. Second, the measure metrics PCC and RMSE were regarded as two main measures for drug-response prediction projects. But different data may have different magnitudes in the drug-response value for their common drugs. Therefore, we can also seek a better measure for drug-response prediction problems.

## Materials and Methods

### Problem Formulation

In this paper, we adopted a powerful collaborative filtering method to predict anti-cancer drug responses in cell lines. The primary idea comes from the basic hypothesis that similar cell lines are sensitive to similar drugs.

Before discussion, we first defined the notational conventions. Each dataset mentioned above is constructed as three matrixes. The response matrix is about *m* cell lines and *n* drugs, arranged in an m×n matrix: $R={\left\{r_{ui}\right\}}_{1\leq u\leq m,1\leq i\leq n}$. The cell-line similarity matrix is about the PCC of gene-expression profile between each of the *m* cell lines, arranged in an m×m matrix: $COEF_{c}={\left\{COEF_{uv}\right\}}_{1\leq u\leq m,1\leq v\leq m}$. The subscript *c* refers to cell line. The drug-similarity matrix is about the Jaccard similarity score of the PubChem fingerprint between each of the *n* drugs, arranged in an n×n matrix: $COEF_{d}={\left\{COEF_{ij}\right\}}_{1\leq i\leq n,1\leq j\leq n}$. The subscript *d* refers to drug. To distinguish between the two similarity matrixes, the special indexing letters are reserved: for cell lines *u* and *v*, and for drugs *i*, *j*, and *k*. Our goal is to predict unknown elements in R based on the known ones, as well as similarity matrixes $COEF_{c}$ and $COEF_{d}$.

NCFGER adopted the typical neighborhood-based CF method for drug-response prediction. The original form, which has been shared by virtually all earlier CF systems, is the user-oriented approach.^31^ Its analogous alternative is the item-oriented approach.^32^ They have been two state-of-the-art techniques for RS. However, the utilization of a user similarity matrix or item similarity matrix only always results in poor prediction accuracy due to the sparseness of preference data. Thus, a hybrid collaborative filtering model is often preferred by combining user-oriented CF and item-oriented CF together. In this way, both user similarity and item similarity are considered for missing value prediction. In this paper, we will focus on the user-oriented approach in the methods introduction section, and simple user-oriented CF (cell-line-based), item-oriented CF (drug-based), as well as hybrid over user-oriented CF and item-oriented CF (hybrid) were all implemented for comparison. The hybrid method simply takes the average of scores predicted from both user-oriented and item-oriented CF methods.

**Figure 7:**
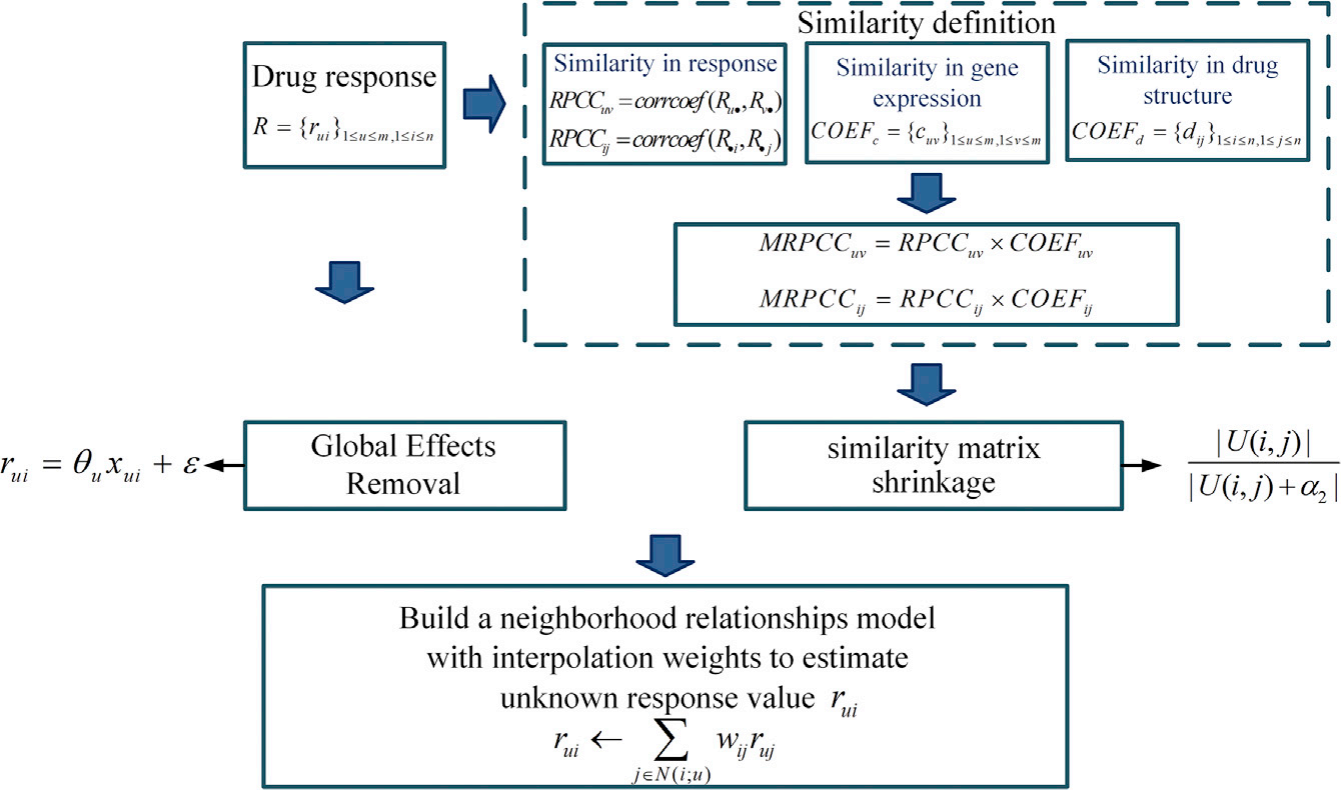
The Workflow of NCFGER

The cell-line-oriented method followed an improved neighborhood based the collaborative filtering method to estimate the unknown response ${\hat{r}}_{ui}$, as is shown in Figure 7. First, there may be large cell line and drug effects—i.e., systematic tendencies for some cell lines to have higher responses than other cell lines and for some drugs to get higher responses than others. This is exactly what we call “global effects” in the RS area. Thus, we adopted the normalization step that helps to remove the “global effects.”

### Preprocessing by Global Effects Removal

Our strategy is to estimate one “effect” at a time, in sequence (i.e., the overall mean of the response IC^50^ value, the main effect of cell line, the main effect of drug, etc.). At each step, we used residuals from the previous step as the dependent variable for the current step. Thus, after each step of effect removal, the $r_{ui}$ refers to residuals, rather than original IC^50^ values.

Let $x_{ui}$ be the explanatory variable of interest corresponding to cell line u and drug i. For cell-line and drug main effects, the $x_{ui}$’s are identically 1. For other global effects, $x_{ui}$ is centered for each cell line by subtracting the mean of $x_{ui}$ for the exact cell line. In each case, the model is defined as

(Equation 1) $$r_{ui}=\theta_{u}x_{ui}+\varepsilon .$$

With sufficient responses for cell line u, the unbiased estimator of θ is

(Equation 2) $${\hat{\theta }}_{u}=\frac{\sum_{i}r_{ui}x_{ui}}{\sum_{i}x_{ui}^{2}}.$$

However, the estimator is somehow unreliable, since some values of ${\hat{\theta }}_{u}$ may be based on very few known responses. To avoid this circumstance, each individual value of ${\hat{\theta }}_{u}$ is shrunk toward a common value from a Bayesian perspective. To be more specific, the true $\theta_{u}$ is supposed to follow a normal distribution. And a slightly simpler estimator used to calculate $\theta_{u}$ is multiplying Equation 2 by the factor defined as

(Equation 3) $$\frac{n_{u}}{n_{u}+\alpha_{1}}$$

where $n_{u}$ is the number of responses of cell line u and $\alpha_{1}$ is a constant, which was set to 3 by cross-validation.

### Similarity Definition

The similarity matrixes are required for identification of *K* nearest neighbors. The original similarity of cell lines $COEF_{c_{uv}}$ is drawn based on the PCC of gene-expression profiles between cell lines *u* and *v*, while that of drug $COEF_{d_{ij}}$ was drawn based on the Jaccard coefficient of drug chemical fingerprint between drugs *i* and *j*. However, to some extent, the similarity between cell lines *u* and *v* can also be shown from the perspective of drug response. Thus, in this paper, we investigated the different similarity definitions for drug-response prediction. To be more specific, the similarity of cell lines can be defined based on gene expression profile’s PCC (*COEF*_*c*_), the correlation coefficient between their response IC^50^ value (*RPCC*_*c*_), as well as the multiplication of *COEF*_*c*_ and *RPCC*_*c*_, which is indicated as *MRPCC*_*c*_ in the following. The exact *MRPCC*_*c*_ similarity measure is defined in Equation 4:

(Equation 4) $$MRPCC_{c_{uv}}=COEF_{c_{uv}}\times RPCC_{c_{uv}}$$

where $RPCC_{c_{uv}}$ is calculated as the PCC between the response IC^50^ values of cell lines *u* and *v*.

In the same way, the similarity between drugs *i* and *j* can be defined based on a drug chemical fingerprint’s Jaccard coefficient (*COEF*_*d*_), the PCC between response IC^50^ values of drugs *i* and *j* (*RPCC*_*d*_), as well as the multiplication of *COEF*_*d*_ and *RPCC*_*d*_ (*MRPCC*_*d*_) defined in Equation 5:

(Equation 5) $$MRPCC_{d_{ij}}=COEF_{d_{ij}}\times RPCC_{d_{ij}}.$$

In order to avoid the bias caused by the different level of support (different number of known responses) for each drug, the similarity matrixes are further shrunk by multiplying $\left|U\left(i,j\right)\right|/\left(\left|U\left(i,j\right)+\alpha_{2}\right|\right)$ for some small $\alpha_{2}$, where U(i,j) is the set of cell lines that have responses to both drugs *i* and *j*. The index of *i* and *j* here will be changed to *u* and *v* for cell lines *u* and *v*. $\alpha_{2}$ was set to 1 by cross-validation in this paper.

### NCFGER

After global effects removal, we can turn to predict the unknown response IC^50^ value for cell line *u* of drug *i*, which is ${\hat{r}}_{ui}$.

Among all drugs that have response values in cell line *u*, we resort to a set of *K* cell lines N(u;i) that tend to have the most similar response in u and that actually have response to drug *i* (i.e., $r_{vi}$ is known for each v∈N(u;i)). *K* is set to 10 in our experiments. The similarity rank is measured based on the shrunk similarity matrix $MRPCC_{c}$ and $MRPCC_{d}$, respectively.

Based on the selected set of *K* neighbors, the interpolation weights $\left\{w_{ij}|j\in N\left(i;u\right)\right\}$, which enable the best prediction of unknown response, can be reached by

(Equation 6) $${\hat{r}}_{ui}\leftarrow \sum_{j\in N\left(i;u\right)}w_{ij}r_{uj}.$$

Thus, $w\;\in R^{K}$. The interpolation weights can be solved by the definition of linear system:

(Equation 7) Aw=b.

It actually models the relationships between drug i and its neighbors through a least-squares problem of

(Equation 8) $$\underset{w}{\min}{\sum_{v\neq u}\left(r_{vi}-\sum_{j\in N(i;u)}w_{ij}r_{vj}\right)}^{2}.$$

For each pair of drugs i and j, we compute

(Equation 9) $${\bar{A}}_{ij}=\frac{\sum_{v\in U\left(i,j\right)}r_{vi}r_{vj}}{\left|U\left(i,j\right)\right|}\;\text{and}\;$$

(Equation 10) $${\bar{b}}_{j}=\frac{\sum_{v\in U\left(i,j\right)}r_{vj}r_{vi}}{\left|U\left(i,j\right)\right|}.$$

Then the best estimator $\hat{A}$ and $\hat{b}$ for *A* and *b* are further improved based on the fact that the averages represented in Equations 9 and 10 may differ by orders of magnitude in terms of the number of cell lines included in the average.

Thus, the corresponding K×K matrix $\hat{A}$ and the vector $\hat{b}\in R^{K}$ is defined as

(Equation 11) $${\hat{A}}_{jk}=\frac{\left|U\left(j,k\right)\right|{\bar{A}}_{jk}+\beta \cdot avg}{\left|U\left(j,k\right)\right|+\beta }\;\text{and}$$

(Equation 12) $${\hat{b}}_{j}=\frac{\left|U\left(i,j\right)\right||{\bar{b}}_{j}+\beta \cdot avg}{\left|U\left(i,j\right)\right|+\beta }$$

where avg denotes a baseline value, which is defined by taking the average of all possible ${\bar{A}}_{jk}$ values. It is obvious that β controls the extent of the shrinkage.

Thus, w’s are achieved by a non-negative quadratic optimization method and are used to predict $r_{ui}$ following Equation 1. The final estimated IC^50^ value of ${\hat{r}}_{ui}$ should be recovered with those removed global effects.

The drug-oriented method is the analog alternative to cell-line-oriented method. Based on the above two methods, the hybrid method got its prediction score based on the mean operation.

## Author Contributions

X.C. conceived the project, designed the experiments, analyzed the results, revised the paper, and supervised the project. L.Z. and H.L. developed the prediction method, designed and implemented the experiments, analyzed the results, and wrote the paper. Y.Z. implemented the experiments, analyzed the results, and revised the paper.

## Conflicts of Interest

The authors have no conflicts of interest.

## Supplemental Information

Document S1. Figures S1 and S2 and Tables S1–S3

Document S2. Article plus Supplemental Information
