# Supplementary material for: Anti-cancer Drug Response Prediction Using Neighbor-Based Collaborative Filtering with Global Effect Removal
Source: Mol Ther Nucleic Acids. 2018 Sep 22;13:303–11. doi: 10.1016/j.omtn.2018.09.011 (PMC6197792; doi:10.1016/j.omtn.2018.09.011)
Supplement: Document S1. Figures S1 and S2 and Tables S1–S3 [file mmc1.pdf]

OMTN, Volume *13*

Supplemental Information

Anti-cancer Drug Response Prediction Using Neighbor-Based Collaborative Filtering with Global Effect Removal

Hui Liu, Yan Zhao, Lin Zhang, and Xing Chen

Supplemental Figures

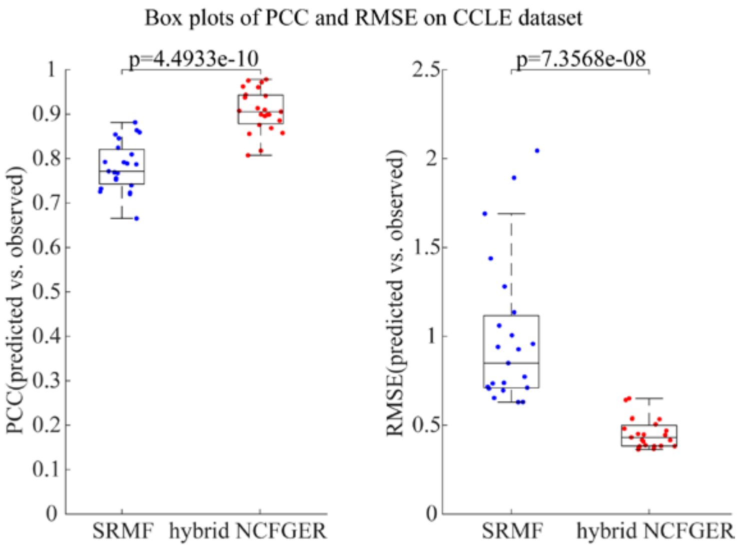

Fig. S1. Box plots of SRMF and our proposed method on CCLE dataset with respect to different evaluation metrics.

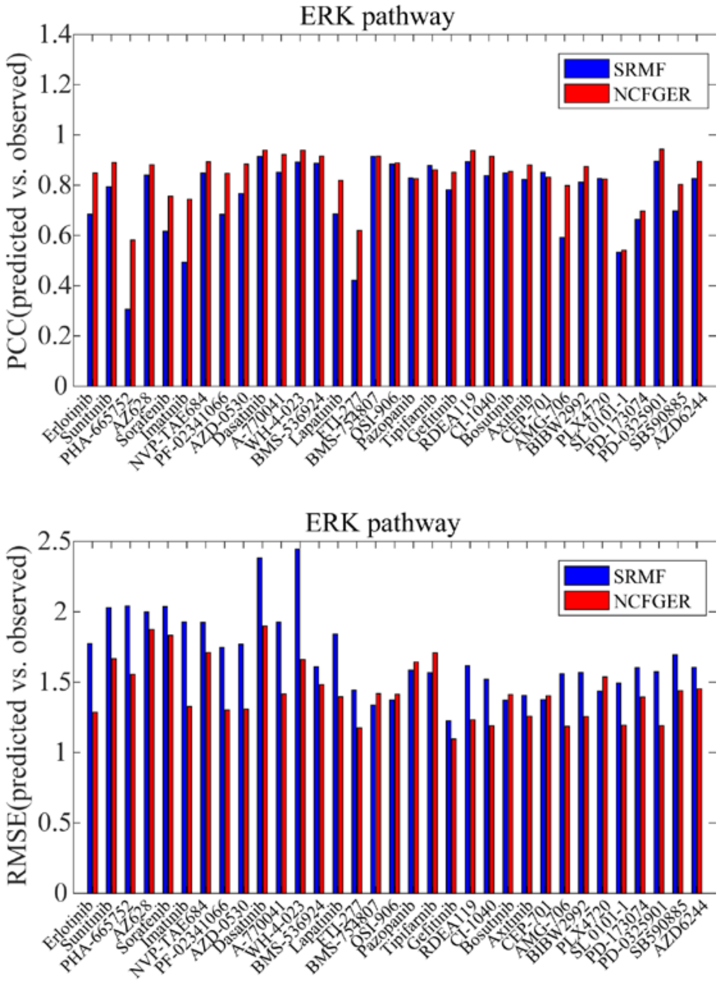

Fig.S2. Prediction performance comparisons of SRMF and NCFGER for the drug targeting genes in ERK pathway with respect to PCC as well as RMSE in CCLE dataset.

Supplemental Tables

Table S1. The comparison results between hybrid NCFGER with different similarity definition and SRMF obtained under 10-fold cross validation on GDSC dataset

Methods		Drug-averaged PCC_S/R	Drug-averaged RMSE_S/R	Drug-averaged PCC	Drug-averaged RMSE
NCFGER	*COEF*	0.78(±0.13)	1.58(±0.34)	0.70(±0.14)	1.27(±0.26)
	RPCC	0.81(±0.11)	1.42(±0.29)	0.73(±0.13)	1.18(±0.24)
	MRPCC	0.81(±0.11)	1.42(±0.29)	0.73(±0.13)	1.18(±0.24)
SRMF		0.71(±0.15)	1.73(±0.46)	0.62(±0.16)	1.43(±0.36)

Table S2. The comparison results between hybrid NCFGER with different similarity definition and SRMF obtained under 10-fold cross validation on CCLE dataset

Methods		Drug-averaged PCC_S/R	Drug-averaged RMSE_S/R	Drug-averaged PCC	Drug-averaged RMSE
NCFGER	*COEF*	0.89(±0.05)	0.47(±0.09)	0.85(±0.07)	0.39(±0.07)
	RPCC	0.91(±0.05)	0.45(±0.08)	0.86(±0.06)	0.38(±0.06)
	MRPCC	0.91(±0.05)	0.45(±0.08)	0.86(±0.06)	0.38(±0.06)
SRMF		0.78(±0.07)	0.74(±0.23)	0.71(±0.09)	0.57(±0.18)

Table S3. The comparison results of both methods obtained under 10-fold cross validation on CCLE dataset

Methods		Drug-averaged PCC_S/R	Drug-averaged RMSE_S/R	Drug-averaged PCC	Drug-averaged RMSE
NCFGER	Hybrid	0.91(±0.05)	0.45(±0.08)	0.86(±0.06)	0.38(±0.06)
	cellline-based	0.85(±0.08)	0.51(±0.06)	0.78(±0.09)	0.47(±0.08)
	Drug-based	0.80(±0.07)	0.63(±0.20)	0.73(±0.09)	0.52(±0.16)
SRMF		0.78(±0.07)	0.74(±0.23)	0.71(±0.09)	0.57(±0.18)
